# Evaluating the Use of Selective Serotonin Reuptake Inhibitors (SSRIs) and Male Infertility: A Critical Retrospective Study

**DOI:** 10.3390/jcm13072129

**Published:** 2024-04-07

**Authors:** Jawza F. Alsabhan, Haya M. Almalag, Lulu A. Alnuaim, Awatif B. Albaker, Maryam M. Alaseem

**Affiliations:** 1 Department of Clinical Pharmacy, King Saud University, P.O. Box 2457, Riyadh 11149, Saudi Arabia; 2 Department of Obstetrics and Gynecology, College of Medicine, King Saud University, P.O. Box 7805, Riyadh 11472, Saudi Arabia; lalnuaim@ksu.edu.sa; 3 Department of Pharmacology and Toxicology, College of Pharmacy, King Saud University, P.O. Box 2457, Riyadh 11495, Saudi Arabia; abaker@ksu.edu.sa; 4 College of Pharmacy, King Saud University, P.O. Box 2454, Riyadh 11451, Saudi Arabia

**Keywords:** antidepressants, infertility, males, semen quality, SSRIs

## Abstract

**Background**: The use of selective serotonin reuptake inhibitors (SSRIs) has been associated with potential effects on male fertility, although the exact mechanisms are not fully understood. The aim of this study was to understand the relationship between SSRIs and male infertility; **Methods**: A retrospective chart review of Saudi males who were treated with SSRIs and attended an infertility clinic in KSMC was undertaken. The medical records of men from an infertility clinic were reviewed to screen the quality of the sperm parameters in patients taking SSRIs; **Results**: In total, 299 men were identified, of whom 29 (9.6%) were exposed to SSRIs, while 270 (90.4%) did not receive SSRIs, defined as the control infertile group. When comparing the mean ages, a notable disparity was observed between the control group of infertile men (34.2 ± 6.9 years) and the infertile group using SSRIs (41.5 ± 3.2 years) (*p* < 0.001). Regarding the sperm analysis and the use of SSRIs, the impact of SSRIs use showed no significant differences in sperm liquefaction (*p* = 0.1), motility (*p* = 0.17), viscosity (*p* = 0.16), or count (*p* = 0.069) with escitalopram, fluoxetine, or paroxetine use; **Conclusions**: Our study showed no significant difference in the sperm analysis between the SSRI and non-SSRI cohorts. However, the relationship between SSRI use and sperm count warrants further investigation and consideration in clinical practice.

## 1. Introduction

Infertility is an essential concern for couples of childbearing age all around the world. It is a disease of the male or female reproductive system, defined by the International Committee for Monitoring Assisted Reproductive Technology (ART) and the World Health Organization (WHO) as the failure to achieve a clinical pregnancy after 12 months or more of regular, unprotected sexual intercourse. The WHO, in conjunction with ART, first listed infertility as a disease in the “International Glossary of ART Terminology” [1].

Infertility is a global issue that affects quality of life and public healthcare and has psychological, financial, and medical consequences [2]. According to recent statistics collected worldwide, approximately 15% of couples who are of childbearing age of both genders are infertile [3]. However, Ombelet et al. revealed that infertility rates could approach 30% in some people in various regions of the world [4]. The prevalence of infertility among couples in Saudi Arabia is approximately 18.93% [5]. However, the absence of an established definition of infertility results in varying estimates of the prevalence of infertility within and among populations. This could be due to the heterogeneous nature of the sampled populations or the denominators used to calculate the prevalence [6]. Unexplained infertility is a term that has been applied to as many as 30–40% of infertile couples and usually refers to a diagnosis or a lack of diagnosis made in couples for whom all the standard investigations, such as ovulation tests, tubal patency, and semen analysis, are normal [7]. Male infertility is characterized by abnormal semen parameters. Hence, semen analysis is the cornerstone of laboratory evaluation of infertile males [8]. Semen analysis remains the single most useful fundamental investigation; with a sensitivity of 89.6%, it can detect 9 out of 10 men with a genuine problem [9]. Various factors beyond lifestyle and environment can contribute to causing male infertility, including habits of cannabis use and exposure to pesticides and heavy metals [10,11]. Furthermore, drugs and medications can influence male fertility by affecting spermatogenesis and sexual dysfunction. Notably, this may be according to five basic mechanisms, including the direct gonadotrophic effect, alteration of the hypothalamic–pituitary–gonadal (HPG) axis, the impairment of ejaculation, erectile dysfunction, and adverse effects on libido [12]. There are various medications with varying degrees of side effects on the male reproductive system, such chemotherapeutics, testosterone, sulfasalazine, anabolic steroids, cyproterone acetate, opioids, tramadol, GHRH analogues, sartans, and hydroxyurea [13,14,15]. However, these were not discussed here because they were beyond the scope of this study. Here, the study focused on and limited the discussion to the effects of anti-depressive medications that may be associated with male infertility, especially selective serotonin reuptake inhibitors (SSRIs). Concerning depressive disorders, according to the National Institute of Mental Health in the USA, in 2020, it was estimated that the highest proportion of adults experiencing a major depressive episode was found among those aged 18 to 25 years, approximately totaling 17.0% of that age group [16]. According to recent statistics, the prevalence of adults with depression in the Kingdom of Saudi Arabia is 32% [17]. The general pattern of antidepressants used in Saudi Arabia is approximately 41.4%; the pattern of SSRI antidepressants used is approximately 37.7%; other classes of antidepressants constitute 18.8%; and tricyclic antidepressants constitute 8.8% [18]. SSRIs, including citalopram, escitalopram, fluvoxamine, paroxetine, fluoxetine, and sertraline, prevent serotonin reuptake. Currently, these drugs are the first-line therapies for anxiety and depressive disorders. However, SSRIs are particularly linked to serious adverse sexual effects, such as reduced libido, a prolonged ejaculatory interval, circulation in erectile dysfunction, and hormones. Research suggests that 25–73% of patients using an SSRI will have some form of sexual dysfunction, which is more severe than that caused by other antidepressants [19]. Recently reviewed research has proven that SSRIs have a detrimental effect on semen quality in in vitro, animal, and human investigations. However, there is currently insufficient evidence to suggest that SSRIs reduce fertility or influence infertility treatment outcomes definitively. However, SSRIs may have an adverse impact on sperm quality [20,21].

In Saudi Arabia, there may also be gaps in our knowledge regarding the use of selective serotonin reuptake inhibitors (SSRIs) and their potential impact on male fertility. While SSRIs are commonly prescribed for the treatment of depression and anxiety disorders, their effects on reproductive health, particularly in males, may not be fully understood within the Saudi population. Research exploring the prevalence of SSRI use among men in Saudi Arabia, as well as the potential associations between SSRI use and male fertility, could help fill these knowledge gaps. 

To sum up, the aim of the study is to investigate the relationship between selective serotonin reuptake inhibitors (SSRIs) and male fertility, specifically focusing on the Saudi Arabian population. This study seeks to address the existing gaps in knowledge regarding the impact of SSRIs on male reproductive health within this demographic. By examining the prevalence of SSRI use among men in Saudi Arabia and assessing its potential effects on sperm parameters, spermatogenesis, and overall fertility outcomes, the study aims to provide valuable insights into the association between SSRIs and male infertility in this context. The findings of this research may inform clinical practice, public health policies, and patient counseling regarding the use of SSRIs and their potential implications for male reproductive health in Saudi Arabia.

## 2. Materials and Methods

### 2.1. Study Design

This observational cross-sectional retrospective study reviewed the medical records of all the men who underwent semen analysis (SA) for fertility evaluation at King Saud Medical City (KSMC). We considered information from all the participants that matched the inclusion criteria. This study used data collected from January 2014 to December 2020. This observational study was guided by the Strengthening the Reporting of Observational Studies in Epidemiology checklist.

### 2.2. Setting

The research setting for this study was KSMC, a tertiary hospital in Riyadh City, which is part of the Ministry of Health in Saudi Arabia. Patients were enrolled from the fertility clinic, including its outpatient offerings. The clinic for newly diagnosed patients was held on 2 days per week on average and received four to eight patients per day. Therefore, the expected total number of patients visiting the clinic per week was approximately 16.

### 2.3. Participants

This study included all the patients who visited the fertility clinic. For the inclusion criteria, based on practical considerations regarding the characteristics of the sample selection, the participants included were adult males aged 26 years and older, in good medical health, and who had at least one semen sample analyzed. Participants using an SSRI class (including citalopram, escitalopram, fluvoxamine, paroxetine, fluoxetine, and sertraline) were compared with participants who did not use SSRIs. The exclusion criteria included male patients diagnosed with infertility due to other problems unrelated to a low sperm count, such as patients who were known or suspected to have any disease-causing hyperprolactinemia, such as hypothalamic–pituitary disease and tumors; patients who were taking medications that influence serotonin receptors other than antidepressants; patients who had prior exposure to spermatotoxic medications, clomiphene citrate, gonadotropins, and selective estrogen receptor modulators; and patients who had artificial urinary sphincters and adjustable male slings [22,23].

### 2.4. Variables, Data Source, and Measurements

The participants’ data were gathered from medical records under the supervision of a consultant, and all the information was collected on a predesigned data collection sheet. The data collection sheet was divided into three sections. The first part concerned the participants’ demographic data, such as age, education level, current occupation, marital status, number of family members, medical and medication history, and smoking status. The second section contained information on the use of SSRIs, such as the drug name, drug dose, frequency, and duration of treatment. The final part of the sheet contained semen analysis information, including physical examination parameters such as volume, color, viscosity, and liquefaction time. The semen analysis included a microscopic examination of sperm motility and morphology. The semen analysis also included a total sperm count. The semen quality of the samples was interpreted according to the Sixth Edition of the WHO Manual for Human Semen Analysis [24].

### 2.5. Macroscopic Examination 

The Supplementary Table provides a comprehensive overview of the parameters related to semen characteristics, including volume, color, liquefaction time, and viscosity. These factors play essential roles in assessing semen quality and male fertility (Table S1).

### 2.6. Microscopic Examination

The Supplementary Table outlines the lower reference ranges for the key parameters related to sperm movement (motility), progression, total motile sperm count, sperm morphology, and sperm count. These parameters are crucial factors in assessing male fertility (Table S2).

### 2.7. Statical Analysis 

The data were analyzed using IBM Statistical Package for Social Sciences version 26 (IBM Corp.: Armonk, NY, USA, 2019) and presented as means (standard deviation) or medians (interquartile range) for continuous variables and numbers or percentages for categorical variables. A bivariate analysis was performed to explore the differences between the patients receiving SSRIs and those not receiving SSRIs in terms of age, smoking status, medical history, and semen analysis. The significance level of the results was set at *p* < 0.05. Binary logistic regression was performed to predict factors contributing to infertility concerning SSRI use. 

## 3. Results

A total of 299 men were identified during their visits to the fertility clinic at KSMC, of whom 29 (9.6%) were exposed to SSRIs, while 270 (90.4%) did not receive SSRIs, defined as the control infertile group. When comparing the results to the mean age group, there was a significant difference between the control group of infertile men (34.2 ± 6.9 years) and the infertile group using SSRIs (41.5 ± 3.2 years), *p* < 0.001. Most of the patients in the control group (n = 168) (62.4%) were nonsmokers, and approximately half of the infertile patients who used SSRIs were similarly nonsmokers (n = 14) (48.2%), with no significant difference (*p* = 0.144). The difference in SSRI use between the control group and infertile men on SSRIs became significant when compared with the mean liquefaction time (*p* = 0.028). Moreover, there was a significant difference in the mean motility (*p* = 0.034) between the infertile group on SSRIs and the control patients. Regarding the semen analysis, macroscopic examination showed that none of the collected samples were statistically significant for either group (*p* = 0.3) (Table 1). For further exploration, regarding the correlation between the age group and the use of SSRIs through odds ratio analysis, the result displayed a significant association (*p* < 0.001), with an odds ratio equal to 1.12 (95% confidence interval [CI] 1.069–1.188), which indicated the patients on SSRIs were older than 40 years, while the non-SSRI patients were approximately 35 years old. In addition, the correlation between the use of SSRIs and sperm motility was statistically significant (*p* < 0.0036). The result revealed that the odds ratio equal to 1.022 for the patients not receiving SSRIs was associated with low motility (less than 40%) (95% C.I 1.001–1.042). However, among the participants who received SSRIs, the odds ratio for a liquefication time of more than 60 min was 0.2 times lower than that in the control group, as indicated by an odds ratio of 0.2 (95% CI: 0.067–1.132) (Table 2).

For the infertile group who received SSRI treatment (n = 29), the most frequently prescribed SSRIs were escitalopram (n = 11, 37.9%) and fluoxetine (n = 11, 37.9%), followed by paroxetine (n = 7, 24.1%). When comparing the results to the mean age group, we found that there was no significant difference among all the SSRIs (*p* = 0.438). Correspondingly, there was no significant difference in the mean SSRI frequency or treatment duration (*p* = 0.21). The impact of SSRI use showed no significant differences in sperm liquefaction (*p* = 0.1), motility (*p* = 0.17), viscosity (*p* = 0.16), or sperm count (*p* = 0.069) with escitalopram, fluoxetine, or paroxetine use (Table 3).

## 4. Discussion 

The study investigated the potential impact of selective serotonin reuptake inhibitors (SSRIs) on the male fertility parameters among participants at the fertility clinic. The findings provide valuable insights into the association between SSRIs and various semen characteristics, shedding light on the potential implications for male reproductive health. Among the 299 men included in the study, 29 (9.6%) were exposed to SSRIs, while 270 (90.4%) were not, forming the control infertile group. The proportion of men exposed to SSRIs in the study was about 9.6%, within the range reported in other research. Studies investigating the prevalence of antidepressant usage among men seeking fertility treatment have reported varying rates, typically ranging from 5% to 15% [20]. 

Our study showed a significant age difference between the SSRI group and the control group, aligning with some previous research [25]. Patients over 40 years old among SSRI users have been consistently reported in studies examining the demographic characteristics of individuals prescribed SSRIs. This suggests that SSRI use may be associated with decreased fertility in older men. Further research is needed to better understand this association. It is possible that SSRI use may also be associated with decreased fertility in younger men.

Furthermore, our result revealed that the smoking status did not significantly differ between the two groups. The similarity in the smoking status between the SSRI group and the control group is consistent with some previous studies, although others have reported higher rates of smoking among individuals with mental health conditions, for which SSRIs are commonly prescribed [26]. 

The study analyzed several semen parameters, including liquefaction time, motility, viscosity, and sperm count. Significant differences were noted in the liquefaction time and motility between the SSRI group and the control group. Specifically, the men in the SSRI group exhibited shorter liquefaction times and a lower sperm motility compared to the control group. However, no significant differences were observed in the macroscopic semen analysis between the two groups. Similarly, another study conducted by Safarinejad et al. revealed the negative effect of SSRIs on semen quality in terms of the sperm count, sperm motility, and morphology with a prolonged duration of treatment [27]. Moreover, four studies comprising 222 male participants were selected for systematic analysis. The results revealed that SSRIs were associated with a reduction in normal sperm morphology (95% CI [−16.29, −3.77], *p* = 0.002), sperm concentration (95% CI [−43.88, −4.18], *p* = 0.02), sperm motility (95% CI [−23.46, −0.47], *p* = 0.04), and the sperm DNA fragmentation index (DFI) (95% CI [6.66, 21.93], *p* = 0.0002). However, there was no statistically significant impact on semen volume (95% CI [−0.75, 0.65], *p* = 0.89) [28]. 

The most prescribed SSRIs were escitalopram, fluoxetine, and paroxetine, with no significant difference in the semen counts among the different prescribed medications. Our results are consistent with a similar previous prospective study that was conducted in 2010 by Tanrikut et al. [29], who examined the adverse effects of paroxetine on semen parameters and sperm DNA fragmentation after 5 weeks of paroxetine administration. The findings were not associated with abnormalities in the other semen parameters [30]. On the other hand, another study linked fluoxetine’s action with gonadotoxic consequences via enhancing sperm concentration and motility, the fragmentation of deoxyribonucleic acid, and the reduced weight of the reproductive organs [20]. 

The duration of SSRI use appears to be a critical factor in understanding its impact on male fertility. Our analysis of the duration of SSRI use revealed varying percentages across different time intervals: less than 2 years, 2–5 years, and more than 5 years. Interestingly, there was a trend towards a higher proportion of participants with longer durations of SSRI use experiencing adverse effects on their fertility parameters, particularly in terms of sperm liquefaction time and motility. Specifically, among the participants with less than 2 years of SSRI use, 50.0% exhibited certain fertility-related issues. This percentage decreased slightly to 25.0% in the 2–5-year duration group. However, in the group with more than 5 years of SSRI use, a substantial 66.7% showed fertility-related issues. These findings suggest a potential cumulative effect of prolonged SSRI use on male fertility, warranting further investigation into the mechanisms underlying this relationship. It is worth noting that while the differences in the fertility parameters across the duration groups did not reach statistical significance (*p* = 0.101), there was a trend towards a higher proportion of long-term users (more than 5 years) in the fluoxetine group compared to the other medications. As a result, the observed trends emphasize the importance of considering the duration of SSRI use in terms of assessing its impact on male fertility. Clinicians should be mindful of this factor when prescribing SSRIs, especially in men of reproductive age, and should weigh the potential risks against the benefits of treatment with SSRIs. In contrast, other studies have suggested the possible negative effects of SSRIs on semen analysis and sperm counts with a prolonged treatment course. A meta-analysis showed that SSRIs negatively affected semen quality [29]. Similarly, another study conducted by Safarinejad et al. revealed the negative effect of SSRIs on semen quality in terms of sperm count, sperm motility, and morphology with a prolonged duration of treatment [27].

Regarding the SSRI dosage and frequency of use, the study revealed that there were no significant differences in the distribution of dosage (*p* = 0.583) or frequency of use (*p* = 0.219) among the users of different SSRIs, indicating similar prescription patterns across the medication groups. This can be explained by healthcare providers often following standardized treatment guidelines when prescribing SSRIs for various psychiatric conditions. These guidelines may recommend specific starting doses and dosing frequencies based on factors such as the severity of symptoms, patient age, and comorbid medical conditions. As a result, clinicians may prescribe similar doses and frequencies of SSRIs regardless of the specific medication being used.

Overall, the data suggest that while there may be variations in the duration of SSRI use among different medications, there are no significant differences in age, smoking habits, dosage, frequency of use, or semen characteristics among users of escitalopram, paroxetine, and fluoxetine. These findings provide valuable insights into the clinical management of psychiatric conditions (depression or anxiety disorders) with SSRIs and underscore the need for personalized treatment approaches based on individual patient factors and treatment goals, especially in male individuals of childbearing age.

The study has many strengths and limitations. One strength is that it is a collaborative study. It involves experts from many specialties. These include an infertility clinic expert and a pharmacist due to the application of multidisciplinary collaboration and comprehensive and well-rounded research. Our study also has practical significance. It provides insight into using SSRIs in policy decisions about male infertility. It covers prescribing and using SSRIs and managing their side effects and future research on male infertility generally. Furthermore, confounding variables such as age, smoking status, and the duration of SSRI use must be considered. Our study was cross-sectional. It could only provide a snapshot of data at a single time. This makes it hard to show the causes of infertility and SSRI use. So, we often need a longer-term study to assess the cause-and-effect links, here between the use of SSRIs and semen analysis. The researchers faced challenges during the data collection due to electronic records missing information. The infertility clinic’s system was manual, and most of the entered data were incomplete. So, the researchers had to refer to the patients’ files and review the data manually. Consequently, the sample size of the patients taking SSRIs was small. Thus, the findings may not apply to the general population. Also, the small sample size in our study lowered its statistical power. This made it hard to detect the effects or the links between SSRIs and semen quality. Additionally, there is a need for randomized clinical trials to support the research findings. Conducting RCTs specifically focused on the effects of SSRIs on male fertility parameters would offer several advantages. Firstly, RCTs allow for the implementation of rigorous study designs with random allocation of the participants into treatment groups, minimizing potential biases and confounding factors. This would enhance the validity and reliability of the findings, providing more robust evidence on the impact of SSRIs on male fertility. Moreover, RCTs enable researchers to directly compare different SSRIs in terms of their effects on fertility outcomes. By including multiple treatment arms representing different SSRIs, researchers can assess whether certain medications have differential effects on semen characteristics, thus providing more nuanced insights into the treatment options for male individuals of childbearing age. Furthermore, RCTs facilitate the implementation of standardized outcome measures and protocols, ensuring consistency across study sites and enhancing the generalizability of the findings. This would allow clinicians to make more informed decisions about SSRI prescribing practices for male patients, considering both psychiatric treatment efficacy and potential impacts on fertility.

## 5. Conclusions

The study suggests that SSRIs may have an impact on certain semen characteristics, particularly liquefaction time and motility, and these effects may vary between different SSRI medications. Clinicians should be mindful of this when considering the treatment options for patients using SSRIs. More research is needed to investigate the potential link between SSRI use and sperm count. We recognize that SSRIs may impact male reproductive health differently. This depends on individual factors, including genetic predispositions, lifestyle habits, and overall health status. It is essential for researchers to conduct more extensive studies before definitive clinical decisions can be made. These future studies should aim to show the mechanisms behind the link between SSRI use and sperm count. Only through rigorous scientific inquiry can a comprehensive understanding and explanation of the implications of SSRI use on male fertility be achieved and informed decisions be made in clinical practice.

## Figures and Tables

**Table 1:** Baseline characteristics of participants with differences between participants receiving SSRI and non-SSRIs.

	Using SSRIs N = 292	%, SD, IQR	Non-SSRIs N = 270	%, SD, IQR	Total N = 299	%, SD, IQR	*p* Value
Age, years, mean (SD)	41.52	3.28	34.20	6.93	34.91	7.00	<0.001 *
Smoking, N (%)							0.144
No	14	7.7	168	92.3	182	60.9	
Yes	15	12.8	102	87.2	117	39.1	
Viscosity, n (%)							0.197
Normal	15	12.4	106	87.6	121	40.5	
Slightly viscous	8	11.3	63	88.7	71	23.7	
Highly viscous	6	5.6	101	94.4	107	35.8	
Liquefaction, N (%)							0.028 *
Less than 30 min	3	17.6	14	82.4	17	5.7	
30–60 min	17	14.4	103	85.8	120	40.1	
More than 60 min	9	5.6	153	94.4	<0.001 * 162	54.2	
Motility, mean (SD)	47.93	11.84	39.55	20.79	40.37	20.23	0.034 *
Sperm count, median (IQR)	20.00	15.00–55.00	29.85	25.00–35.00	28.80	24.30–33.75	0.300

* Significant according to a *p*-value < 0.050; SSRIs, selective serotonin reuptake inhibitors; SD, standard deviation; IQR, upper and lower interquartile range.

**Table 2:** Baseline characteristics and infertility-related information stratified by SSRI medication.

	Escitalopram N = 11	%, SD, IQR	Paroxetine N = 7	%, SD, IQR	Fluoxetine N = 11	%, SD, IQR	Total N = 29	%, SD, IQR	*p* Value
Age, years, mean (SD)	40.55	3.24	41.71	3.35	42.36	3.32	41.52	3.28	0.438
Smoking, N (%)									0.418
No	4	28.6	3	21.4	7	50.0	14	48.3	
Yes	7	46.7	4	26.7	4	26.7	15	51.7	
Dose, N (%)									0.583
10 mg	2	40.0	0	0.0	3	60.0	5	17.2	
20 mg	7	36.8	5	26.3	7	36.8	19	65.5	
40 mg	2	40.0	2	40.0	1	20.0	5	17.2	
Frequency, N (%)									0.219
Once daily	9	39.1	4	17.4	10	43.5	23	79.3	
Twice daily	2	33.3	3	50.0	1	16.7	6	20.7	
Duration of use, N (%)									0.101
Less than 2 years	4	50.0	2	25.0	2	25.0	8	27.6	
2–5 years	3	20.0	3	20.0	9	60.0	15	51.7	
More than 5 years	4	66.7	2	33.3	0	0.0	6	20.7	
Viscosity, n (%)									0.167
Normal	7	46.7	4	26.7	4	26.7	15	51.7	
Slightly viscous	3	37.5	0	0.0	5	62.0	8	27.6	
Highly viscous	1	16.6	3	50.0	2	33.3	6	20.7	
Liquefaction, N (%)									0.100
Less than 30 min	3	100.0	0	0.0	0	0.0	3	10.3	
30–60 min	6	35.3	3	17.6	8	47.1	17	58.6	
More than 60 min	2	22.2	4	44.4	3	33.3	9	31.0	
Motility, mean (SD)	44.55	11.93	45.00	12.25	53.18	10.55	47.93	11.84	0.177
Sperm count, median (IQR)	20.00	15.00–45.00	10.00	4.00–60.00	44.00	13.00–90.00	20.00	15.00–55.00	0.069

SD, standard deviation; IQR, upper and lower interquartile range; mg, milligram.

**Table 3:** Odds ratio of participants receiving SSRI with regards to sperm characteristics.

	B	S.E	*p*-Value	Odds Ratio
Age	0.119	0.027	<0.001 *	1.127
Motility	0.021	0.010	<0.036 *	1.022
Liquification 30–60 min	−0.261	0.010	0.074	0.770
Liquification > 60 min	−1.293	0.723	0.074	0.275
	B	S.E	*p*-value	Adjusted £ Odds ratio
Motility	0.024	0.011	0.029 *	1.025
Liquification 30–60 min	0.072	0.725	0.921	1.075
Liquification > 60 min	−1.102	0.761	0.148	0.332

£ adjusted for age; * significant at *p* < 0.050.

## Data Availability

The data presented in the study are available on request from the corresponding author.

## Supplementary Materials

The following supporting information can be downloaded at https://www.mdpi.com/article/10.3390/jcm13072129/s1. Table S1: The supplementary table provides a comprehensive overview of the parameters related to semen characteristics, including volume, color, liquefaction time, and vis-cosity. Table S2: The supplementary table outlines the lower reference ranges for the key parameters related to sperm movement (motility), progression, total motile sperm count, sperm morphology, and sperm count.

## Abbreviations

The following abbreviations are used in this manuscript:

ART	Assisted Reproductive Technology
PR	Progressive motility
SSRIs	Selective serotonin reuptake inhibitors
SA	Semen analysis
WHO	World Health Organization
