# Supplementary material for: Evaluating the Use of Selective Serotonin Reuptake Inhibitors (SSRIs) and Male Infertility: A Critical Retrospective Study
Source: J Clin Med. 2024 Apr 7;13(7):2129. doi: 10.3390/jcm13072129 (PMC11012779; doi:10.3390/jcm13072129)
Supplement: Supplementary file 1 [file jcm-13-02129-s001.zip › jcm-2885993-supplementary.pdf]

Supplementary online only material

Table S1

Parameter	Lower Reference Range	Description
**Volume**	≥ 1.5 mL	The lower reference limit for semen volume; low volume may indicate pathological conditions or collection problems.
**Color**	Whitish to gray	Normal color of semen; red-brown appearance may indicate blood presence; yellow color may indicate jaundice or medication side effects.
**Liquefaction**	15–30 min	Time taken for semen to liquefy; necessary for sperm movement; delay can affect fertility.
**Viscosity**	Normal	Normal semen viscosity shows small discrete drops; high viscosity can affect sperm motility and concentration.

Table S2

Parameter	Lower Reference Range
**Total Motility**	42%
**Progressive Motility (PR)**	30%
**Total Motile Sperm Count**	39 million
**Normal Sperm Morphology**	4%
**Sperm Count**	15 million/mL
